# Managing right-sided colon cancer in the frail patient

**DOI:** 10.1007/s00464-024-11417-2

**Published:** 2024-11-19

**Authors:** T. Shakir, G. Lingam, N. Francis

**Affiliations:** 1 University College London, London, UK; 2 St Marks Hospital and Academic Institute, London, UK; 3 The Griffin Institute, Harrow, UK

The concept of performing a simple right hemicolectomy has traditionally been considered a straightforward operation, in order to start ones learning curve with respect to Minimally Invasive Surgery (MIS); this no longer seems the case following alarming reports demonstrating worse oncological outcomes when compared to left sided resections or even rectal cancer [1–3]. Although this can be attributed to a number of factors, quality of surgery has been the center of this debate. Earlier reports demonstrated a 27% survival benefit in stage III disease, for those patients who had surgery along the mesocolic plane [4], prior to the first report of the concept of complete mesocolic excision (CME) in by Hohenberger in 2009 [5]. This technique involves meticulous dissection along embryological planes to remove the mesocolon intact, with central ligation of the vascular pedicle to maximize the harvest of lymphovascular structures, linked to accurate staging and reducing the risk of local 5-year recurrence [3].

Since then, this technique has received significant attention from the colorectal community globally and generated evidence to examine its efficacy using various approaches, including laparoscopic and robotic approaches.

Neither the indications, nor surgical technique, of CME have been firmly defined in literature. The indication for CME is not routinely discussed as part of the multi-disciplinary team meeting and is often left to the discretion of the surgical team to decide whom to offer this technique, resulting in a significant heterogeneity of practice regarding the application of this technique among the global colorectal community. This is often underpinned by the availability of surgical expertise to perform such a procedure, in addition to patient factors, such as tumor demography and co-morbidities. Given the availability of expertise, anecdotally, CME is often offered to more advanced tumors, affecting the transverse colon, and in younger and fitter patients [6].

Many surgeons are reluctant to routinely offer CME for right-sided colon cancer, as this is often influenced by the perceived feeling among surgeons as more challenging. This is supported by some evidence that MIS, especially laparoscopic CME, is associated with longer operative times and increased perioperative risks [3, 7]. The RELARC randomized trial reported a significant difference in vascular injury with CME, although no overall difference in short term complications [7]. It is likely that these risks would be exacerbated in frail patients, who have diminished physiological reserves and are less able to tolerate the stress of prolonged surgery. Furthermore, the true effect size may be overestimated, with a systematic review highlighting median locoregional recurrence in non-metastatic colon cancer with CME was > 0.5% [8].

In the era of rising uptake of robotic surgery, CME seems to present a feasible and acceptable option which is preferred by many surgeons for right-sided colonic cancer due to the steady platform, enhanced 3D magnified views, improved dexterity and ergonomics. It is arguably easier to robotically dissect around the superior mesenteric vein as well as the trunk of Henle, compared to laparoscopic approach.

These robotic benefits undoubtedly, have expanded the indications and application of CME to a wider patient context. Previous surgery, high body mass index and older patients groups, in whom laparoscopic CME may have not been considered, are now more amenable to a robotic approach.

## Should CME be offered to elderly or frail patients?

As technology and techniques of surgery and perioperative care progress, we tend to offer radical surgery to a far older population then before. When elderly patients are considered, a clear distinction should be made between chronological and biologic age. Age alone should not be the determining factor. Although elderly patients are likely to be more comorbid than their younger counterparts, frailty should be assessed in its own right and be separated from elderly. Thus, frail patients require a more nuanced approach to their care. Pre-habilitation programs are now well established to optimize the physiological and psychological well-being of these patients prior to surgery. Several reports have supported its success in improving outcomes, as well as converting those patients who were deemed to be unsuitable for major surgery such as CME, by improving the anaerobic threshold during cardiopulmonary exercise testing [9–12].

When frail patients are offered CME, however, the balance between the potential oncological benefits versus the additional risk associated with CME surgery, must also be measured. Longer operative times are associated with poorer rates of 5-year survival and disease-free survival [13]. Other factors should be considered especially when MIS CME is offered, such as the possible impact of prolonged peritoneal insufflation and patient positional change, particularly in the context of prior cardio-respiratory co-morbidities. Similarly, the concept of MIS should also be carefully considered with this group of patients and often the advantages of MIS can be critical to those patients with respiratory disease. Careful consideration with the anesthetist is required.

Additionally, one must be careful to offer such an advanced approach as estimates of life expectancy are reported to be between 1.2 and 3.4 years after a diagnosis of frailty [14]. While achieving an acceptable oncological outcome is of paramount importance, the increased perioperative risks in frail patients necessitate careful assessment of overall health and ability to withstand surgery. Furthermore, CME aims to enhance the quality of surgery and optimize staging but one must be cognoscente of the physical burden of chemotherapy if postoperative adjuvant therapy is required. Therefore, we must weigh the benefits versus the risks of extensive resections, such as CME, especially if they are performed using MIS against the increased perioperative risks in frail patients. In some cases, a less extensive resection may be warranted to reduce operative time and minimize complications for those frail patients.

Nevertheless, treatment decisions such as CME must be carefully considered for all patients and explored at the tumor board or Multidisciplinary Team Meeting (MDT). This will ensure equity of care and deliver fair and equal opportunities to all patients. In order to achieve this, prior knowledge of patient functional status and co-morbidities should be available at the MDT discussion to support appropriate decision making. This will also require input from anesthetic and pre-assessment teams who are not regularly part of the colorectal MDT. Therefore, there is a call to expand the MDT function to include further discussion of selected patients with a wider group of perioperative care, including anesthetic, cardiology, and/or geriatric physician input to discuss the pros and cons of such extensive surgery. This comprehensive MDT will require full preoperative objective functional assessment, frailty scoring (as measured by the functional status of patients using tools, such as the World Health Organization score), the Comprehensive Geriatric Assessment, as well as the outcome of any cardiopulmonary exercise testing. Surgical expertise to undertake this procedure is essential to ensure safe performance, particularly when minimally invasive surgery (MIS) is considered. Ultimately, patient choice is fundamental and this requires patient education and information about various modalities of surgical techniques, presenting the risks and benefits of this radical surgery versus less radical approaches.
