# Correction: Managing right-sided colon cancer in the frail patient

**DOI:** 10.1007/s00464-024-11444-z

**Published:** 2024-12-02

**Authors:** T. Shakir, G. Lingam, N. Francis

**Affiliations:** 1 University College London, London, UK; 2 St Marks Hospital and Academic Institute, London, UK; 3 The Griffin Institute, Harrow, UK

**Correction to: Surgical Endoscopy** 10.1007/s00464-024-11417-2

The original online version of this article was revised to correct a sentence in the third paragraph of the introductory section. The corrected sentence reads:

Neither the indications, nor surgical technique, of CME have been firmly defined in the literature.

The original article has been corrected.

